# The relative atrial volume ratio and late gadolinium enhancement provide additive information to differentiate constrictive pericarditis from restrictive cardiomyopathy

**DOI:** 10.1186/1532-429X-13-15

**Published:** 2011-02-25

**Authors:** Huaibing Cheng, Shihua Zhao, Shiliang Jiang, Minjie Lu, Chaowu Yan, Jian Ling, Yan Zhang, Qiong Liu, Ning Ma, Gang Yin, Renate Jerecic, Zuoxiang He

**Affiliations:** 1 Department of Radiology, Cardiovascular Institute and Fuwai Hospital, Chinese Academy of Medical Sciences and Peking Union Medical College, Beijing 100037, PR China; 2 Department of Nuclear medicine, Cardiovascular Institute and Fuwai Hospital, Chinese Academy of Medical Sciences and Peking Union Medical College, Beijing 100037, PR China; 3 MR Research and Development, Siemens Medical Solutions USA, Inc., Chicago, IL, USA

## Abstract

**Background:**

The differentiation of constrictive pericarditis (CP) from restrictive cariomyopathy (RCM) is often difficult. This study sought to determine the clinical utility of cardiovascular magnetic resonance imaging (CMR) for differentiating both these disorders.

**Methods:**

Twenty-three patients with surgically documented CP, 22 patients with RCM and 25 normal subjects were included in the study. CMR yielded information about cardiac morphology, function and tissue characteristics. The left (LA) and right atrial (RA) volume was calculated using the area-length method. The relative atrial volume ratio (RAR) was defined as the LA volume divided by RA volume. Receiver operating characteristic curve analysis was used to test the ability of different variables in differentiating CP from RCM.

**Results:**

The maximal pericardial thickness in CP patients was significantly larger than in normal subjects and RCM patients. The RA volume index in RCM patients (90.5 ± 35.3 mL/m^2^) was significantly larger than in CP patients (71.4 ± 15.7 mL/m^2^, p = 0.006) and normal subjects (38.1 ± 9.0 mL/m^2^, p < 0.001). The LA volume index in RCM (96.0 ± 37.0 mL/m^2^) and CP patients (105.6 ± 25.1 mL/m^2^) was significantly larger than in normal subjects (39.5 ± 9.5 mL/m^2^, p < 0.001 for all). The RAR in CP patients (1.50 ± 0.29) was significantly larger than in RCM patients (1.12 ± 0.33, p < 0.001) and normal subjects (1.06 ± 0.20, p < 0.001). There were no differences between RCM patients and normal subjects in the RAR (p = 0.452). At a cut-off value of 1.32 for the RAR, the sensitivity was 82.6%, and the specificity was 86.4% in the detection of CP. Septal bounce was identified in 95.7% CP patients, in none of RCM patients and normal subjects. Late gadolinium enhancement (LGE) was present in 31.8% RCM patients and absence in all CP patients and normal subjects.

**Conclusions:**

CMR with LGE and RAR can facilitate differentiation of CP from RCM.

## Background

Clinical and hemodynamic features are often similar in constrictive pericarditis (CP) and restrictive cardiomyopathy (RCM), but differentiation of these 2 conditions is crucial because CP requires surgical treatment and is usually curable, while RCM, short of cardiac transplantation, is treatable only by medical means and often responds unsatisfactorily [1-3]. At present, clinical evaluation, measurement of pericardial thickness, analysis of septal motion, quantitative assessment of systolic and diastolic myocardial function, invasive pressure measurement, and endomyocardial biopsy have been useful in this differential diagnosis, but no one diagnostic method can be relied upon to make the distinction by itself [4-7].

Cardiovascular magnetic resonance (CMR) provides high-resolution imaging of the pericardium and associated structures in any imaging plane. It fuses excellent anatomic detail and tissue characterization with accurate evaluation of cardiac function and assessment of the haemodynamic consequences of pericardial constraint on cardiac filling [8-12]. Compared with echocardiography and computed tomography, CMR with late gadolinium enhancement (LGE) is the only method that can depict the presence of myocardial fibrosis, which may well facilitate diagnosis of RCM resulting from infiltrative myocardial disease and have important prognostic implications [13-17].

The aim of the present study was to describe the clinical utility of CMR for distinguishing CP form RCM. We sought to determine the diagnostic accuracy of the relative atrial volume ratio (RAR) for the detection of CP and its possible use as a screening tool to aid in the differentiation between CP and RCM.

## Methods

### Study population

The study population consists of 45 consecutive patients who were referred for CMR, including 23 surgically documented CP patients and 22 RCM patients. All patients had been underwent previously systematic clinical evaluation, including history and examination, electrocardiography, chest radiography, and echocardiography. In each CP case, surgical confirmation was obtained by the presence of an obliterated pericardial space, an adhesive pericarditis with bulging of the heart out of the pericardial incision at pericardiectomy and pathological confirmation. The diagnosis of RCM was confirmed by pathological specimens or based on impaired cardiac filling (i.e., increased filling pressures and no echo-Doppler evidence of respiratory-dependent ventricular coupling) in combination with pericardial thickness < 2 mm. All patients were referred to rule out any other cardiovascular diseases such as coronary artery disease, hypertension, valvular and congenital heart disease, and other cardiomyopathy. As a control group, 25 normal subjects without a history of cardiovascular symptoms or risk factors were also included in this study.

The study was approved by the institutional ethics committee, and all subjects gave written informed consent.

### CMR protocol

Cardiac magnetic resonance imaging was performed in all patients by using a 1.5-T unit (Magnetom Avanto; Siemens Medical Solutions, Erlangen, Germany) with a high-performance gradient system (maximum gradient amplitude 45 mT/m; maximum slew rate 200-μs rise time), a 12-element-body phased-array coil system and electrocardiographic triggering. The CMR examinations began with the acquisition of survey images in three orthogonal planes (transverse, coronal, and sagittal) to localize the heart within the chest. Next, we studied the heart by performing a dark blood half-Fourier acquisition single-shot turbo spin echo (HASTE: repetition time [TR]/echo time [TE] = 700/26 ms, slice thickness = 6 mm, flip angle = 160°, field of view [FOV] = 350 mm) and turbo spin-echo (TSE) T1- (TR/TE = 700/20 ms, slice thickness = 6 mm, flip angle = 180°, matrix = 256 × 156; FOV = 350 mm) and T2-weighted (TR/TE = 800/77 ms, slice thickness = 6 mm, matrix = 256 ×190, FOV = 350 mm, flip angle = 180°) CMR sequences. Left ventricular (LV) short-axis, horizontal long-axis, and vertical long-axis views were used to evaluate cardiac function on cine CMR sequences. Cine CMR were acquired using generalized autocalibrating partially parallel acquisitions (GRAPPA: TR/TE = 45.9/1.07 ms, slice thickness = 6 mm, matrix = 109 × 192, FOV = 350 mm, flip angle = 80°) or time-adaptive sensitivity encoding (TSENSE: TR/TE = 41.7/1.39 ms, slice thickness = 6 mm, matrix = 109 × 192, FOV = 350 mm, flip angle = 70°) with true fast imaging with steady-state precession (TrueFISP) cine sequences. 15 to 20 minutes after injection of 0.2 mmol/kg of gadolinium diethylenetriamine pentaacetic acid (Magnevist, Schering, Berlin, Germany), the images of LGE were obtained in standard short axis covering the entire ventricle, and in long axis views to detect areas of LGE using a phase-sensitive inversion recovery (PISR) spoiled gradient echo sequence (TR/TE = 8.7/3.4 ms, slice thickness = 6 mm, imaging matrix = 256 × 256, FOV = 350 mm, flip angle = 15°).

### CMR Analysis

**Figure 1:**
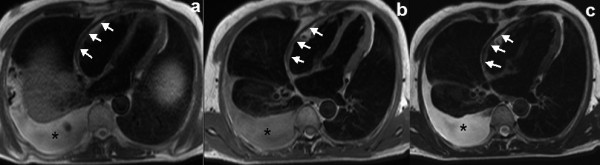
**Diffuse thickened pericardium**. HASTE (A), T1- (B) and T2-weighted (C) TSE images showed diffuse pericardial thickening (white arrows) which is most pronounced over the RV and RA and moderate right-sided pleural effusion (*).

All CMR images were transferred to workstation (Siemens medical systems) for analysis. Qualitative assessments were performed independently by three readers. If there was a discrepancy, majority opinion was used. Quantitative measurements were performed independently by two readers. All observers were blinded to the diagnosis. For morphological evaluation of the pericardium, TSE and HASTE images were employed (Figure 1) to assess the maximum pericardial thickness. Septal motion was evaluated on a short-axis cine function view 1 cm beneath the atrioventricular valves on a visual basis and described as normal and the early diastolic septal bounce. The biventricular volumes and ejective fraction were obtained using Argus analytical software (version VE36A). Endocardial margins of the LV and right ventricular (RV) were semi-automatically contoured on end-diastolic and end-systolic images. End-diastolic and end-systolic frames were defined on the basis of the respective image frames demonstrating the largest and smallest cavity size. For the left atrial (LA) volume, the biplane area-length method was used. For the right atrial (RA) volume, the monoplane area-length formula was used. Atrial diastole was determined by selecting the last frame in ventricular systole before mitral valve opening. The measurements were made according to published methods [18]. The long-axis length of the LA and RA was defined by measuring the distance from the center of the mitral annulus to the posterior atrial wall. The atrial endocardial area was manually traced to exclude the atrial appendages and pulmonary or caval veins. Body weight and body height were measured and the body surface area was calculated. Subsequently, division with body surface area indexed all CMR variables apart from the ejection fraction. The RAR was defined as the LA volume divided by RA volume. LGE was considered present only if myocardial enhancement was confirmed on both short-axis and matching long-axis locations using a signal intensity threshold of > 2 standard deviation (SD) above a remote reference region in the same image.

### Statistical analysis

All values were given as mean ± SD or counts (percentage). Categorical values were compared by chi-square test or Fisher exact test as appropriate. Comparisons of normally distributed continuous variables between the different groups were performed by using one-way analysis of variance (ANOVA) with Fisher's least significant difference (LSD) posttest. The Kruskal-Wallis and Mann-Whitney U test were used to compare non-normally distributed continuous variables. The inter-observer agreement for the RAR was tested using intra-class correlation coefficient and limits of agreement using Bland Altman plots. Receiver operating characteristic (ROC) curve analysis was used to test the ability of different variables in differentiating CP from RCM. The area under the ROC curve (AUC) for each variable was calculated and compared. The statistical comparison of the ROC curves was performed using MedCalc (11.4.4, MedCalc, Belgium). Other statistical analyses were performed using SPSS for Windows (version 16.0; SPSS Inc., Chicago, IL, USA). P values < 0.05 were considered significant.

## Results

### Patient characteristics

There were 18 men and 5 women in CP group, with a mean age of 43.0 ± 20.2 years (range 15 to 77 years). The aetiology of CP was unknown in 10 patients, whereas 4 patients had previous cardiac surgery, 7 had tuberculous infection, and 2 had history of an inflammatory infection. The RCM group included 22 patients (12 men, 10 women) with a mean age of 47.5 ± 18.5 years (range 14 to 72 years). Five RCM patients underwent heart transplantation, and surgical pathology specimens showed the presence of cardiac amyloidosis in 3 patients and nonspecific findings in 2 patients. Endomyocardial biopsy was performed in other 10 RCM patients. Cardiac amyloidosis was confirmed in 2 patients, mixed connective tissue disease in 1 patient, and idiopathic forms in 7 patients. There were no significant differences between RCM patients and the two other groups in terms of gender, age or BSA distribution. The demographic and clinical characteristics in each group and their comparison are shown in Table 1.

**Table 1:** Baseline and clinical characteristics

Variable	RCM	CP	Normal
Number	22	23	25
Male (n, %)	12 (54.5)	18 (78.3)	14 (56)
Age (yrs)	47.5 ± 18.5	43.0 ± 20.2	42.4 ± 11.2
Height (cm)	167.1 ± 8.4	173.7 ± 23.9	167.8 ± 7.0
Weight (kg)	63.9 ± 11.2	61.9 ± 7.9	64.5 ± 8.1
Base surface area (m^2^)	1.71 ± 0.16	1.72 ± 0.13	1.73 ± 0.11
Symptom			
Dyspnea (n, %)	13 (59.1)	14 (60.9)	0
Edema (by history) (n, %)	14 (63.6)	11 (47.8)	0
Palpitations (n, %)	7 (31.8)	4 (17.4)	0
Fatigue (n, %)	10 (45.5)	9 (39.1)	0
Orthopnea (n, %)	6 (27.3)	5 (21.7)	0
Physical examination			
Jugular venous distension (n, %)	14 (63.6)	17 (73.9)	0
Pulmonary rales (n, %)	6 (27.3)	4 (17.4)	0
Hepatosplenomegaly (n, %)	5 (22.7)	5 (21.7)	0
Ascites (n, %)	3 (13.6)	4 (17.4)	0
Lower-extremity edema (n, %)	12 (54.5)	11 (47.8)	0
NYHA functional class	1.8 ± 1.1	2.1 ± 1.1	1

### CMR characteristics

**Figure 2:**
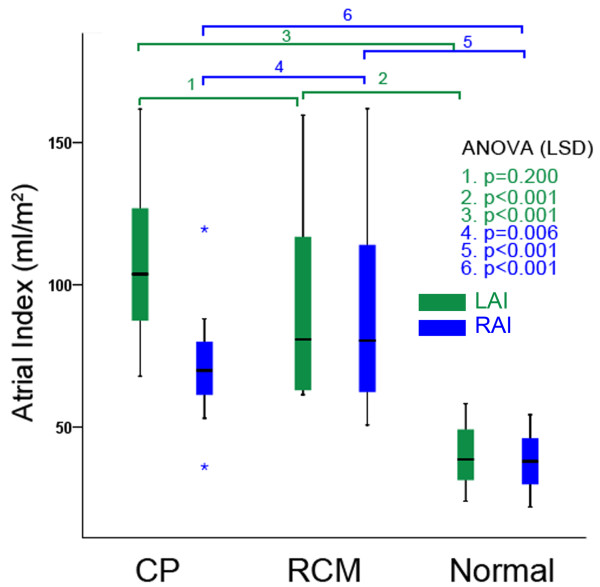
**Left and right atrial volume indices**. Comparisons of LAI and RAI between CP, RCM patients and normal subjects.

**Figure 3:**
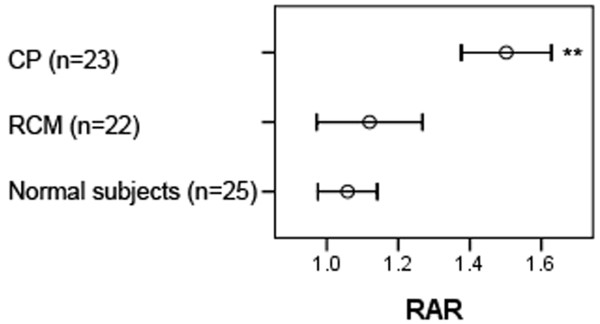
**Error bar of the RAR**. Data are presented as means (squares) and 95% confidence interval (whiskers). **p < 0.001.

**Figure 4:**
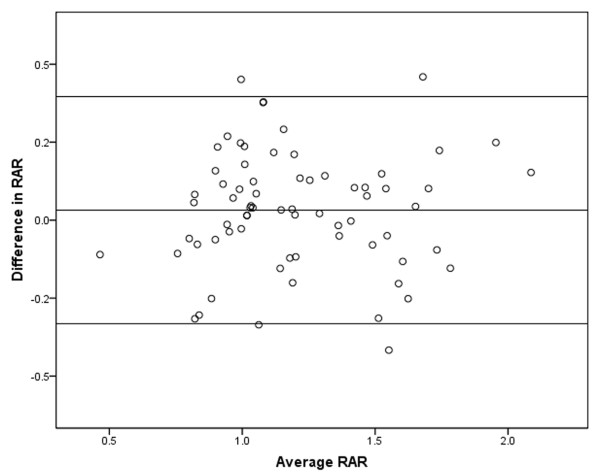
**Bland Altman plot of the RAR values**. Bland-Altman analysis showed excellent inter-observer agreement for the RAR.

The maximal pericardial thickness in CP patients (6.9 ± 2.6 mm, range 4-12 mm) was significantly larger than in normal subjects (1.5 ± 0.4 mm, range 0.9-2.7 mm, p < 0.001) and RCM patients (2.0 ± 0.7 mm, range 1.0-3.4 mm, p < 0.001). There were no differences among the three groups in biventricular end-systolic volume index. There were no differences between CP and RCM patients in biventricular end-diastolic volume index, stroke volume index, and EF, although these values were significantly smaller in RCM and CP patients compared with normal subjects. The RA volume index (RAI) in RCM patients (90.5 ± 35.3 mL/m^2^) was significantly larger than in CP patients (71.4 ± 15.7 mL/m^2^, p = 0.006) and normal subjects (38.1 ± 9.0 mL/m^2^, p < 0.001). Although the LA volume index (LAI) yielded significantly increased values in RCM (96.0 ± 37.0 mL/m^2^) and CP patients (105.6 ± 25.1 mL/m^2^) compared with normal subjects (39.5 ± 9.5 mL/m^2^, p < 0.001 for all), no statistical significance were reached between CP and RCM patients (p = 0.200) (Figure 2). The RAR in CP patients (1.50 ± 0.29) was significantly larger than in normal subjects (1.06 ± 0.20, p < 0.001) and RCM patients (1.12 ± 0.33, p < 0.001). There were no differences between RCM patients and normal subjects in the RAR (p = 0.452) (Figure 3). The intra-class correlation coefficient [equal to 0.917 with 95% confidence interval (CI), 0.84-0.92] showed that there was an excellent inter-observer agreement on the measurement of the RAR. The Bland-Altman plots showing the limits of agreement are shown in Figure 4.

**Figure 5:**
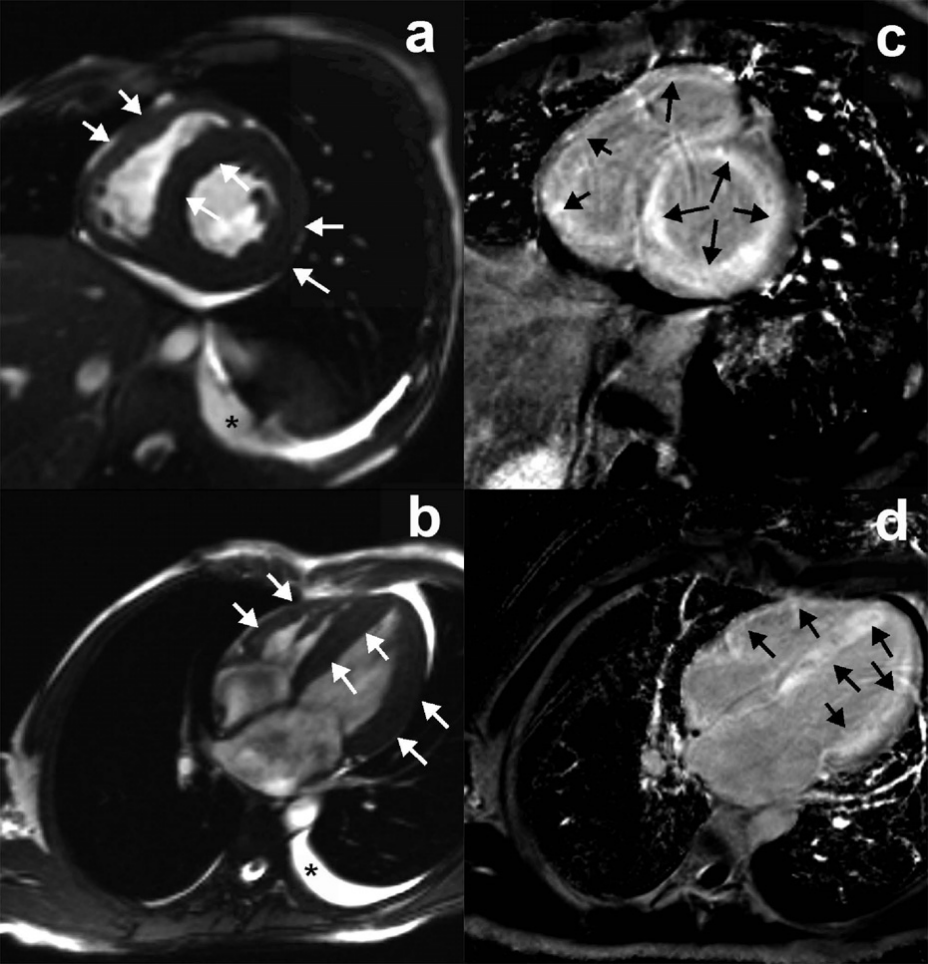
**LGE of cardiac amyloidosis**. CMR demonstrated global LV and RV wall hypertrophy (a and c) (white arrows), diffuse transmural LGE (b and d) (black arrows), and mild left-sided pleural effusion (*) in a 39-year-old male patient with cardiac amyloidosis who underwent cardiac transplantation and was proven by surgical pathology specimen.

The analysis of septal movement during early diastole revealed a septal bounce in 22 CP patients. One CP patient, all RCM patients and normal subjects had a normal septal configuration during diastole. Comparative results of CMR parameters are independently shown in Table 2. LGE was present in 7 of 22 RCM patients (31.8%) and absence in all CP patients and normal subjects. Several different patterns of LGE were present in RCM patients. In 4 of 5 patients with histopathologically proven cardiac amyloidosis, LGE was distributed over the entire subendocardial circumference, extending in various degrees into the neighboring myocardium and the papillary muscles. In remaining 1 patient with cardiac amyloidosis, diffuse transmural LGE was found in the LV wall (Figure 5). Two idiopathic RCM patients had focal areas of LGE in various locations of the LV myocardium.

**Table 2:** Cardiac magnetic resonance imaging findings

Variable	RCM (n = 22)	CP (n = 23)	Normal (n = 25)
Pericardial thickness (mm)	2.0 ± 0.7	6.9 ± 2.6*	1.5 ± 0.4* ^#^
LAI (mL/m^2^)	96.0 ± 37.0	105.6 ± 25.1	39.5 ± 9.5* ^#^
RAI (mL/m^2^)	90.5 ± 35.3	71.4 ± 15.7*	38.1 ± 9.0* ^#^
RAR	1.12 ± 0.33	1.50 ± 0.29*	1.06 ± 0.20**^#^**
LVEDV index (mL/m^2^)	65.4 ± 22.8	60.4 ± 15.0	81.0 ± 12.9* ^#^
LVESV index (mL/m^2^)	36.2 ± 19.9	34.1 ± 13.6	33.2 ± 8.1
LVSV index (mL/m^2^)	29.1 ± 9.9	26.2 ± 9.0	47.7 ± 8.5* ^#^
LVEF (%)	46.6 ± 11.8	44.2 ± 12.4	59.1 ± 6.4* ^#^
RVEDV index (mL/m^2^)	66.8 ± 19.9	59.0 ± 13.1	81.3 ± 12.8* ^#^
RVESV index (mL/m^2^)	37.3 ± 18.1	32.7 ± 10.9	31.6 ± 7.1
RVSV index (mL/m^2^)	29.5 ± 9.3	26.4 ± 9.6	49.7 ± 9.0* ^#^
RVEF (%)	45.8 ± 11.9	44.7 ± 12.9	61.1 ± 6.3* ^#^
Septal bounce, %	0	22 (95.7)*	0* ^#^
LGE (n, %)	7 (31.8)	0*	0* ^#^

LAI = left atrial volume index; RAI = right atrial volume index; RAR = relative atrial volume ratio; EDV = end-diastolic volume; ESV = end-systolic volume; SV = stroke volume; EF = ejective fraction; Comparison with RCM *P < 0.05; Comparison with CP ^#^P < 0.05

### Sensitivity and specificity

**Figure 6:**
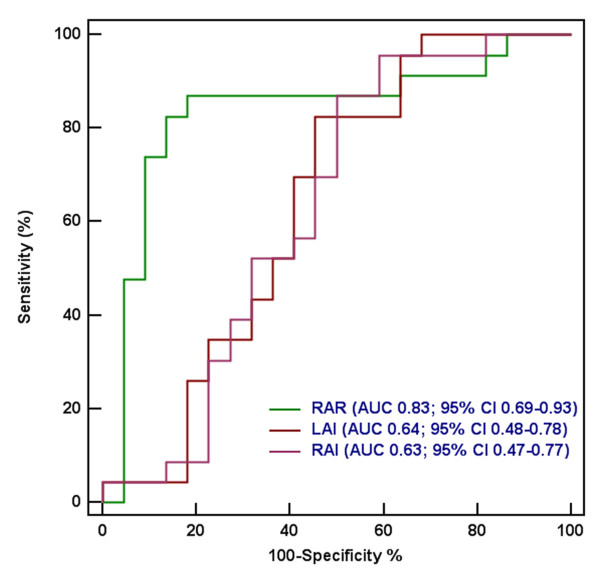
**Receiver operating characteristics curves**. ROC curves described the performance of different variables in differentiating CP from RCM.

Receiver operating characteristic curve analysis was used for comparison of discriminative capacity between different indices (Table 3 and Figure 6). The RAR [AUC 0.83 (95% CI 0.69-0.93)] had higher accuracy than LAI [AUC 0.64 (95% CI 0.48-0.78), p = 0.0216] and RAI [AUC 0.63 (95% CI 0.47-0.77), p = 0.0378] for predicting CP. There were no differences between the LAI and RAI (p = 0.950) in identifying CP. At a cut-off value of 1.32 for the RAR, the sensitivity was 82.6%, and the specificity was 86.4% in the detection of CP. Cut-offs of LAI > 83.4 mL/m^2 ^and RAI ≤ 81.1 mL/m^2 ^had sensitivities of 82.6% and 54.6%, respectively, and specificities of 87.0% and 50.0%, respectively.

**Table 3:** Diagnostic accuracy of individual parameters to distinguish between CP and RCM

Variable	Cut-off Value	AUC (95%CI)	Sensitivity	Specificity
LAI	83.42 mL/m^2^	0.638 (0.482 - 0.776)	82.61(61.2 - 95.0)	54.55(32.2 - 75.6)
RAI	81.14 mL/m^2^	0.628 (0.472 - 0.768)	86.96(66.4 - 97.2)	50.0(66.4 - 97.2)
RAR	1.32	0.834 (0.693 - 0.928)*	82.61(61.2 - 95.0)	86.36(65.1 - 97.1)

AUC = area under receiver operating characteristics curve; 95% CI = 95% confidence interval; other abbreviations as in Table 2; *Statistically significant, p < 0.001

## Discussion

Both RCM and CP are often characterized by normal or decreased volume of both ventricles associated with biatrial enlargement, normal LV wall thickness and atrioventricular valves, impaired ventricular filling with restrictive physiology, and normal (or near normal) systolic function. Echocardiography, computed tomography, CMR and invasive cardiac catheterization have been useful in this differential diagnosis [4-7,10,12,13], but the diagnosis remains equivocal after extensive testing in a subset of patients.

The principal finding of this study demonstrated that the RAR was higher in CP patients than in RCM patients. The pathophysiological hallmarks of pericardial constriction, which are caused by confinement of the cardiac chambers by the rigid, fixed pericardial volume, are limitation of outward expansion of cardiac chambers. The pericardial oblique sinus lies behind the LA so that the posterior wall of the LA is actually separated from the pericardial space. Compared with the RA, the outward expansion of the LA may be less limited by the rigid and fixed pericardium in CP patients, which can lead to out-of-proportion expansion of the LA and RA. In RCM patients, the restrictive physiology caused by decreased myocardial compliance affects both ventricles, while the normally compliant pericardium allows for significantly prominent expansion of the LA and RA at the same time. In this study, the RAR in CP patients was significantly larger than in normal subjects and RCM patients. There were no differences between RCM patients and normal subjects in the RAR. The AUC of RAR was greater than those of the other parameters, while the AUC between the LAI and RAI did not show a difference. These results suggest that the RAR is a useful index for differentiating CP from RCM. These findings are of clinical significance, as substantial differentiation between CP and RCM could not be often made from extensive clinical and noninvasive testing.

The early diastolic septal bounce, a brief rapid motion of the ventricular septum toward the RV in early diastole, is considered a reliable echocardiopraphic and CMR sign of pericardial constriction [1,12]. As shown in other studies as well as herein, abnormal diastolic septal bounce had a sensitivity of 96%, a specificity of 100% for the prediction of surgically proven CP.

The versatility of CMR for CP not only enables accurate, noninvasive, quantitative and qualitative assessment of the pericardium and its associated structures, but also facilitates differentiation from a restrictive physiology that could be challenging clinically. These findings consist of a thickened, fibrotic, and/or calcified pericardium, a sigmoid-shaped septum, a restrictive filling pattern with an enhanced early filling, a respiratory-related variation in the position of the interventricular septum, and an extension of the fibrocalcific process into the underlying myocardium [8,10-13,19]. Moreover, CMR is helpful by its ability to characterize tissues, especially the demonstration of interstitial or nodular fibrosis based on the underlying etiology. The recent studies have showed that CMR has been used to characterize the type of infiltrative RCM by the location and distribution of LGE and may well facilitate diagnosis of RCM resulting from infiltrative myocardial disease, for example, cardiac amyloidosis [14-17]. In patients with systemic amyloidosis, LGE is highly sensitive and specific for the identification of cardiac involvement. Ruberg FL et al.[17] demonstrated that the sensitivity, specificity, positive predictive value and negative predictive value of LGE for the identification of clinical cardiac involvement was 86%, 86%, 95%, and 67% respectively. In the group with histologically proven cardiac amyloidosis, our CMR findings are in line with the recent report. Five RCM patients with LGE were histologically proven cardiac amyloidosis, 4 of whom had the entire subendocardial circumference enhancement. Vogelsberg H et al. [14] reported that patients with biopsy-proven cardiac amyloidosis had a distinct pattern of LGE, which was distributed over the entire subendocardial circumference, extending in various degrees into the neighboring myocardium. They concluded that using this pattern as a diagnostic criterion, the sensitivity of CMR for diagnosing cardiac amyloidosis was 80%, yielding a specificity of 94%. The positive predictive value was 92%, and the negative predictive value was 85%.

In addition, CMR techniques might be used to study the hemodynamic. Francone M et al. [13] reported that real-time cine CMR can easily depict increased ventricular coupling, which may be helpful to better differentiate between CP and RCM patients, especially in patients with normal or minimally thickened pericardium. Recently, Bauner K et al. [20] reported that velocity-encoded flow measurements with calculation of transtricuspid e- to a-wave ratios are a valuable tool for detection of diastolic dysfunction in CP patients.

## Study limitations

The study is limited by a small sample size, which may cause a statistical bias, and larger numbers of patients should be addressed in a future study. RCM patients shared a lot of characteristics with CP patients. In this study, we applied strict standardized diagnostic criteria of RCM. However, histological proof was not available for all RCM patients, and therefore, CP patients with normal pericardial thickness may have been included in RCM patients [21].

## Conclusions

In conclusion, CMR has the potential to enable precise assessment of morphology, function, and tissue characteristics of the heart, which can facilitate differential diagnosis between CP and RCM. If the differential diagnosis between CP and RCM could not be made from extensive clinical and noninvasive testing, a further analysis of both the RAR and LGE is helpful in distinguishing CP from RCM.

## List of abbreviations used

(CP): Constrictive pericarditis; (RCM): Restrictive cardiomyopathy; (CMR): Cardiovascular magnetic resonance; (LGE): Late gadolinium enhancement; (RAR): Relative atrial volume ratio; (LV): Left ventricular; (RV): Right ventricular; (LA): Left atrial; (LAI): Left artial volume index; (RA): Right atrial; (RAI): Right atrial index; (SD): Standard deviation; (ROC): Receiver operating characteristics; (AUC): Area under receiver operating characteristics curve; (CI): Confidence interval.

## Competing interests

The authors declare that they have no competing interests.

## Authors' contributions

HC drafted both the text and figure file. SZ and SJ conceived and designed this study. JR and JL revised the manuscript. ML, CY and YZ were involved with data acquisition. ZH and NM helped draft the manuscript. GY and QL assisted with data analysis and statistics. All authors take responsibility for the entire content of this study and have read and approved the submission of this manuscript.
